# Development and evaluation of a virtual reality mechanical ventilation education program for nursing students

**DOI:** 10.1186/s12909-022-03834-5

**Published:** 2022-11-10

**Authors:** Hanna Lee, Jeong-Won Han

**Affiliations:** 1 Department of Nursing, Gangneung-Wonju National University, Gangwon-do, South Korea; 2 College of Nursing Science, Kyung Hee University, 26, Kyunghee-daero, Dongdaemun-gu, Seoul, 02453 South Korea

**Keywords:** Education, Mechanical ventilation, Virtual reality

## Abstract

**Background:**

Since COVID-19 limits safe clinical practice settings, virtual reality (VR) emerged as an alternative to practical education. Using VR simulation to learn mechanical ventilation is rare in nursing education.

**Methods:**

This study developed a VR simulation program for mechanical ventilation care and evaluated its effects. We adopted a quasi-experiment design. Participants were fourth-year students across nursing colleges in South Korea.

**Results:**

The interaction effect of the intervention time point and control group, to which the VR simulation program was applied, showed a significant difference in self-efficacy (F = 19.54, *p* < .001) and clinical reasoning capacity (F = 16.97, *p* < .001). Learning satisfaction of the experimental group was statistically significantly higher than that of the control group(F = 5.22, *p* = .026). The level of learning immersion (t = − 3.13, *p* = .003) and learning satisfaction (t = − 3.49, *p* = .001) were statistically significantly higher in the experimental group than in the control group.

**Conclusion:**

These findings confirmed that the VR stimulation program for nursing students had a positive effect on their self-efficacy, clinical reasoning capacity, and learning satisfaction. Furthermore, it was effective in improving their nursing competence.

## Introduction

Symptoms of COVID-19 have a diverse clinical course, ranging from asymptomatic infection to severe pneumonia and death. Dyspnea, hypoxia, or infiltration of more than 50.0% of the lungs occur in approximately 14.0% of all confirmed cases. Furthermore, severe symptoms, such as respiratory failure, shock, and multiple organ damage, occur in approximately 5.0% [1]. The most significant complication in patients critically ill with COVID-19 is acute respiratory distress syndrome (ARDS) [2]. ARDS is acute hypoxia with an arterial partial pressure (PaO_2_)/inspiratory oxygen fraction (FIO_2_) ratio of less than 300 mmHg. Patients with ARDS receive respiratory assistance through mechanical ventilation in the intensive care unit (ICU) [3, 4].

Mechanical ventilation nursing, a complex and dynamic process compared to other nursing practices, requires extensive knowledge and skills to ensure safe breathing treatment and reduce complications [5]. In particular, since inappropriate mechanical ventilation management poses an immediate threat to the patient’s life, nurses’ role in mechanical ventilation is critical [6]. Furthermore, it is important for nurses to acquire the knowledge and skills to apply mechanical ventilation appropriately in various situations since the health condition of patients who are critically ill can change frequently [7]. It is impossible for nurses to understand and provide mechanical ventilation nursing in a short time as it requires repeated and systematic education and practice [5]. Therefore, it is necessary for nursing students, future healthcare providers, to systematically learn mechanical ventilation in school.

However, due to the recent spread of COVID-19 and concerns regarding patient safety, nursing students have limited clinical practice [8]. Furthermore, education and clinical experience in mechanical ventilation acquired in the ICU are also relatively low. This indicates the unmet social demand for an increase in the number of skilled nursing professionals for patients who are critically ill. New nurse graduates face considerable stress and confusion when performing mechanical ventilation nursing for patients who are acutely ill [9]. According to previous study [10], since the ability to cope with various situations was important in ventilator nursing, it was necessary to develop educational programs to enhance mechanical ventilation nursing competency. Therefore, finding an effective method of mechanical ventilation nursing education for nursing students is necessary.

Virtual reality (VR) is a technology that enables users to experience real-world experiences through artificial sensory stimulation without directly experiencing the environment or situation [11, 12]. Since it has become difficult to ensure a safe clinical practice setting due to COVID-19, VR has emerged as an alternative to practical education. Its advantage is that students can participate in a safe environment without time and place restrictions [13]. Practical education using VR content also allows non-invasive work at a low cost, with no concern regarding the risk of patient safety incidents that may occur during clinical practice. Moreover, it enables repeated learning in an environment similar to an actual clinical setting [14]. In particular, studies that applied VR to nursing education showed positive effects on nursing performance, critical thinking, problem-solving ability, self-efficacy, and learning immersion required in mechanical ventilation nursing [15–17]. In the educational field, there are many limitations related to direct patient nursing practice during COVID-19 pandemic. Hence, the use of VR simulation as an educational method is likely to have a positive effect [4].

The ongoing COVID-19 pandemic makes it difficult to reproduce mechanical ventilation nursing in education via existing methods for patients who are critically ill. Therefore, we considered the increasing social demand for nurturing nursing personnel for patients who are critically ill with COVID-19, the limited clinical practice of nursing students, and the necessity of repeated learning of realistic mechanical ventilation nursing in safe practice environment. Hence, this study aimed 1) to develop a mechanical ventilation nursing program using VR and 2) evaluate the effect of knowledge, self-efficacy, clinical reasoning capacity, learning immersion, and learning satisfaction among nursing students. The hypothesis of this study is as follows. 1) There will be a difference in the knowledge for mechanical ventilation nursing between the experimental group to which the mechanical ventilation nursing VR simulation program is applied and the control group. 2) There will be a difference in the self-efficacy for mechanical ventilation nursing between the experimental group and the control group. 3) There will be differences in the clinical reasoning competency for mechanical ventilation nursing between the experimental group and the control group. 4) There will be differences in the learning immersion between the experimental group and the control group. 5) There will be differences in the learning satisfaction between the experimental group and the control group.

## Material and methods

### Study design

This study adopted a quasi-experiment design (nonequivalent control group and non-synchronized pretest-posttest design).

### Participants

**Fig. 1:**
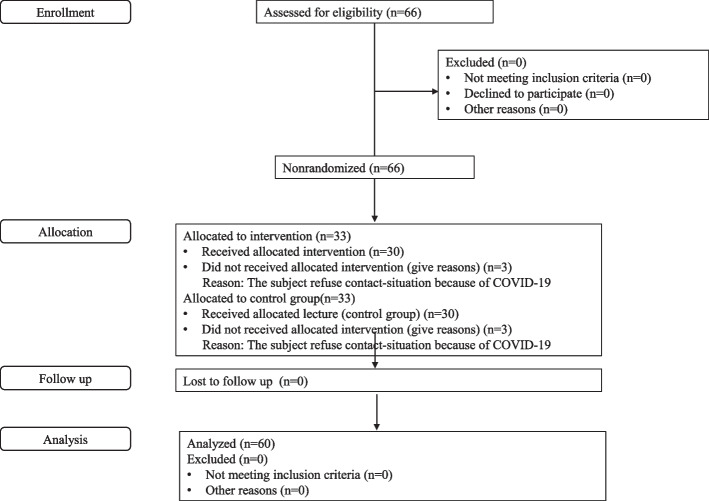
Research subjects

The participants were students from nursing colleges in South Korea. Participation inclusion criteria were students who provided informed consent to participate and exclusion criteria were students who had previously experienced mechanical ventilation nursing VR simulation practice. In addition, participants with a high risk of health problems that could be caused by wearing VR goggles were excluded. The sample for this study was calculated using the G*Power 3.1.2 program [18] with a two-tail test for the difference between the two independent means (two groups), 1:1 assignment, a power of .80, a significance level of .05, and an effect size of .80. The minimum sample size for each group was 26. Based on the effect size for knowledge (effect size immediately after intervention: d = 1.13 and after 2 months: 0.75) measured as a main variable in a previous study [19] that applied a VR simulation program based on knowledge, also a variable in this study, the effect size was set at (f) = .80 (large). We considered the ongoing social distancing due to COVID-19 and selected 66 students as the study participants (33 people per group), considering a 20.0% dropout rate (Fig. 1). Since three students from each group were excluded due to quarantine, there were 30 students each in the intervention and control groups.

### Measurements

#### Knowledge of mechanical ventilation nursing

In this study, the knowledge of mechanical ventilation comprised 10 items developed by researchers based on references. The content validity was verified by three nursing professors, who lectured on the respiratory system and intensive care nursing, and two nurses with more than a decade of experience in the ICU. Each correct answer was scored as 1, and each incorrect answer was scored as 0; a higher total score indicated a higher level of knowledge related to mechanical ventilation. The reliability of the scale was 0.70 based on Kuder-Richardson formula 20 (KR-20).

#### Self-efficacy for mechanical ventilation nursing

Self-efficacy for mechanical ventilation nursing was measured using a scale developed by Ha and Koh [9], which comprised 10 items on the level of confidence in knowledge, attitude, and skills for nursing patients to whom mechanical ventilation was applied. Each item was scored on a 0–10-point scale (0 = “not at all confident” to 10 = “very confident”), and a higher score indicated a higher level of self-efficacy for mechanical ventilation nursing. Cronbach’s ⍺‘s were 0.97 and 0.78 in Ha and Koh’s study [9] and this study, respectively.

#### Clinical reasoning capacity

Clinical reasoning capacity was measured using a 15-item scale scored on a 5-point Likert scale developed by Liou et al. [20]. The scale was translated into Korean and verified by Joung and Han [21]. A higher score indicated a higher degree of clinical reasoning competency. Cronbach’s α’s were 0.94, 0.93, and 0.96, in Liou et al’s study [20], Joung and Han’s study [21], and this study, respectively.

#### Learning immersion

The level of learning immersion in nursing students was measured using the Flow Short Scale developed by Engeser and Rheinberg [22] and translated into Korean and verified by Yoo [23]. The scale comprised 10 items measured on a 5-point Likert scale. A higher score indicated a higher degree of learning immersion. Cronbach’s α’s were 0.92, 0.84, and 0.94 at the time of development in Yoo’s study [23], and this study, respectively.

#### Learning satisfaction

Learning satisfaction was measured using the Numeral Rating Scale (NRS), scored as 10 points for “very satisfied” and 0 points for “very unsatisfied,” and a higher score indicated a higher level of satisfaction with learning.

### Program development process

**Fig. 2:**
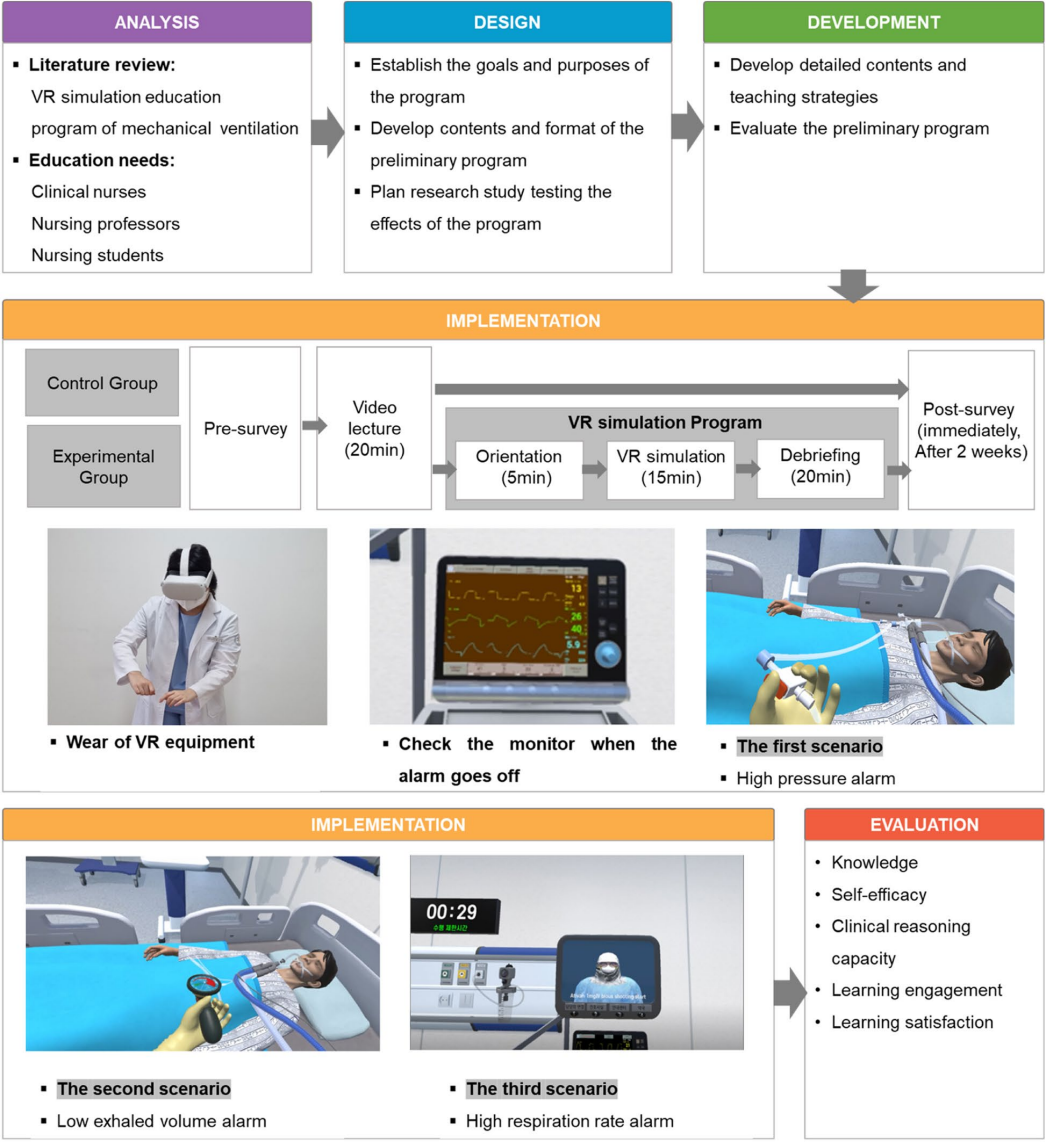
Program development

The program development in this study was conducted using the ADDIE (analysis, design, development, implementation, and evaluation) model [24] (Fig. 2).

#### Analysis

The analysis stage involved acquiring basic data necessary to develop VR simulation scenarios and educational programs. For this, interviews were conducted through an educational needs survey with an expert group, which consisted of three nurses, two nursing school professors, and two nursing college students with experience in intensive care, as well as a literature review on VR simulation related to mechanical ventilation. Through the literature review and interviews, the VR experience education content necessary for nursing students was defined as nursing necessary for coping with the alarms of patients to whom machine ventilation was applied. Additionally, the operating hours of the VR program and contents necessary for pre-learning and debriefing were confirmed.

#### Design

This study was conducted on patients diagnosed with ARDS, who were critically ill, as a complication of confirmed COVID-19. The learning goals were: 1) students could check the alarms of patients who had applied mechanical ventilation to determine what the problem was 2) and provide the appropriate care accordingly. The algorithms for nursing to cope with the alarms of patients to whom mechanical ventilation was applied were set and comprised three algorithms: high pressure alarm, low exhaled volume alarm, and high respiration rate alarm. When the alarm sounded, the program enabled appropriate care after the monitor was checked, and the three modules were displayed randomly. Nursing actions that could be performed within the program included lung sound auscultation, intra-tracheal pressure check, patient condition assessment, ventilation line connection, suction care, doctor’s notification, and administration nursing. The program consisted of pre-learning, orientation, VR simulation, and debriefing.

#### Development

In the development stage, algorithms and scenarios related to nursing to be performed on patients on mechanical ventilation were developed. A prior learning curriculum for acquiring related knowledge before performing VR simulation was composed. To review the VR simulation algorithm and scenario composition, the composition of pre-learning, and the suitability and applicability of the learning goals and opinions were collected from three nurses with experience in ICUs and two nursing college professors. The content validity index (CVI) of the experts was confirmed to be 0.8 or higher for all items. Before the developed program was applied to the participants, its potential as a nursing education program was reviewed by applying a simulation to one clinical nurse to assess if there were any difficulties in understanding the educational contents. The final VR simulation program was developed after the problems were corrected and supplemented after another preliminary survey to assess if there was any additional educational content required. Thereafter, two nursing students and researchers conducted a mock operation three times to increase the fidelity of the research. The VR simulation program was divided into three phases, and consisted of an orientation (5 minutes), VR simulation (15 minutes), and debriefing (20 minutes). The VR simulation operation time was 15 minutes considering the opinions of professors of nursing department and VR production experts who had operated VR systems and that wearing Oculus goggles for a long time may reduce the effect of learning along with eye fatigue.

#### Implementation (intervention)

The experimental and control groups took a video lecture. The lecture was pre-recorded so that consistent content could be delivered to the participants regardless of who gave the lecture, when the lecture was given, or who were the participants. In the experimental group, after examining the video lecture, a trained research assistant conducted an orientation on the VR program and equipment for one person in the experimental group prior to the simulation for VR experience. The debriefing allowed the participants to check the contents of nursing performance expected from the learner, centered on the algorithm of the scenario after the VR experience, whereas, the control group took only the video lecture. The experimental and control groups were separated from each other.

#### Evaluation

In both the intervention and control groups, a preliminary survey was conducted on the general characteristics, knowledge of mechanical ventilation nursing, self-efficacy, clinical reasoning capacity, and learning immersion before they proceeded to the VR program and lectures. For both groups, a post-survey was conducted on the knowledge of mechanical ventilation nursing, self-efficacy, clinical reasoning capacity, learning immersion, and learning satisfaction after the VR program and lecture. Two weeks later, the post-survey was conducted among the participants again.

### Data collection and analysis

Data collection was conducted from December 20, 2021 to April 25, 2022. The author provided contact information in the recruitment notice to allow interested participants to contact the author directly. Afterwards, a trained research assistant explained the study’s purpose and process to each participant and obtained written informed consent. For data analysis, SPSS version 23.0 for Windows was used, and the Shapiro-Wilk test was performed to evaluate the normality of the variables prior to the program’s application. As prior homogeneity tests for the general characteristics and variables of the participants, Chi-squared tests, Fisher’s exact tests, and independent t-tests were conducted. After the intervention, a two-way repeated measures analysis of variance, repeated measure ANOVA, paired t-test, and two-sample t-test were performed to check the effect of programe.

### Ethical consideration

This study was approved by the Institutional Review Board (IRB) of the institution to which the author belongs.

## Results

### Homogeneity of the subject and pre-dependent variables

There were no factors that showed a statistically significant difference in the homogeneity test between the intervention and control groups according to the participants’ general characteristics (Table 1). In this study, the pre-dependent variables, which included knowledge of mechanical ventilation nursing, self-efficacy, and clinical reasoning competency, were confirmed to be homogeneous with the assumption of normality in both the intervention and control groups (Table 2).

**Table 1:** Homogeneous test of general characteristics of the participants

						(*N* = 60)
Variables	Category	Control (*n* = 30)		Experimental (*n* = 30)		χ^2^ or t (*p*)
		n or Mean	% or SD	n or Mean	% or SD	
Gender	Male	3	5.0	1	1.7	1.07(.61)^a^
	Female	27	45.0	29	48.3	
Age (yr)		22.8	1.51	22.2	0.85	2.00(.051)
Religion	Yes	8	13.3	7	11.7	0.09(.76)
	None	22	36.7	23	38.3	
Satisfaction of clinical practice	Satisfied	28	46.6	28	46.6	0.00 (1.00)^a^
	Dissatisfied	2	3.4	2	3.4	
Satisfaction of major	Satisfied	29	48.3	28	46.6	0.35(.99)^a^
	Dissatisfied	1	1.7	2	3.4	
Satisfaction of college	Satisfied	29	48.3	28	46.6	0.35(.99)^a^
	Dissatisfied	1	1.7	2	3.4	

*SD* Standard deviation

^a^Fisher’s exact test

**Table 2:** Homogeneous test of dependent variable

					(*N* = 60)
Variables	Control (*n* = 30)		Experimental (*n* = 30)		t (*p*)
	Statistics^a^ (*p*)	M ± SD	Statistics^a^ (*p*)	M ± SD	
Knowledge	0.94(.106)	7.17 (1.81)	0.93(.050)	7.67 (1.58)	−1.12(.269)
Self-efficacy	0.97(.453)	3.54 (1.47)	0.97(.500)	3.15 (1.77)	0.92(.371)
Clinical reasoning capacity	0.98(.877)	2.63 (0.60)	0.95(.158)	2.38 (0.69)	1.47(.147)

*M* Mean, *SD* Standard deviation

^a^Shapiro-wilk test

### Effectiveness of the VR simulation program

#### Knowledge

The effect between the groups was not statistically significant. The effect by time point within group was statistically significant, and the interaction effect between the groups and time point (F = 0.02, *p* = .881) was not statistically significant (Table 3).

**Table 3:** Effect of VR simulation programe

2Variables	Experimental (*n* = 30)	Control (*n* = 30)	t*(p* value^b)^	F*(p* value^c^) for interaction
	Mean ± SD	Mean ± SD		
***Knowledge***				0.02(.881)
Pre-test	7.67 ± 1.58	7.17 ± 1.81	−1.12(.269)	
Post-test 1	8.47 ± 1.36	8.17 ± 1.36	−0.85(.410)	
Post-test 2	8.57 ± 3.63	7.97 ± 1.54	−0.82(.414)	
*p* value^a^	.007	.007		
***Self-efficacy***				19.54(<.001)
Pre-test	3.15 ± 1.77	3.54 ± 1.47	0.92(.361)	
Post-test 1	7.18 ± 1.67	5.98 ± 1.39	−2.99(.004)	
Post-test 2	7.28 ± 1.77	5.41 ± 1.06	−3.74(<.001)	
*p* value^a^	<.001	<.001		
***Clinical reasoning capacity***				16.97(<.001)
Pre-test	2.38 ± 0.69	2.63 ± 0.60	1.47(.147)	
Post-test 1	3.91 ± 0.51	3.49 ± 0.60	−2.89(.006)	
Post-test 2	3.90 ± 0.64	3.31 ± 0.78	−3.11(.003)	
*p* value^a^	<.001	<.001		
***Learning immersion***				
Post-test	4.18 ± 0.47	3.68 ± 0.73	−3.13(.003)	N/A
***Learning satisfaction***				
Post-test	8.80 ± 1.24	6.90 ± 2.05	−4.32(<.001)	N/A

*N/A* Not applicable

*p* value^a^ for within group comparisons were computed using repeated measures ANOVA

*p* value^b^ for between group comparisons were computed using independent t-test

*p* value^c^ for between group comparisons at baseline were computed using t-way repeated measures ANOVA

#### Self-efficacy

The effect between the groups and effect by time point were statistically significant. The interaction effect between the groups and time point (F = 19.54, *p* < .001) was statistically significant (Table 3).

#### Clinical reasoning capacity

The effect between the groups and effect by time point were statistically significant. The interaction effect between the groups and time point (F = 16.97, *p* < .001) was statistically significant (Table 3).

#### Learning immersion

The level of learning immersion was statistically significantly higher in the experimental group than in the control group (t = − 3.13, *p* = .003) (Table 3).

#### Learning satisfaction

The level of learning satisfaction was statistically significantly higher in the experimental group than in the control group (t = − 3.49, *p* = .001) (Table 3).

## Discussion

First, the knowledge of mechanical ventilation nursing in the experimental group, to whom the mechanical ventilation nursing VR simulation program was applied, and control group was significantly different based on the time point. However, there was no significant difference in the interaction effect between the groups and time point. This result was consistent with that of a previous study [25] which reported that there was no significant difference in knowledge scores between the intervention group, who received VR simulation, and the control group who studied with textbook data in neuroanatomy education for medical students [25]. This finding was also inconsistent with another study that reported that the knowledge of the experimental group was higher than that of the control group after educational materials using VR related to chemotherapy administration was provided to nursing students in Taiwan [11]. The video lecture conducted in this study seemed helpful in improving the theoretical knowledge of both the intervention and control groups. In particular, the knowledge of both groups may not have differed significantly as the knowledge measurement scale simply measured whether their knowledge was correct or not. However, considering previous research results [26] that VR education was effective in delivering procedural knowledge to students when repeated self-directed learning lasted 30 minutes and an intermediate level of learning immersion were provided, increasing the number of learning sessions is necessary to verify the effectiveness of the program in future research.

Second, a hypothesis of this study, “there would be a difference in the self-efficacy for mechanical ventilation nursing between the experimental group, to whom the mechanical ventilation nursing VR simulation program was applied, and the control group,” was supported by the findings, which was consistent with the results of a previous study [27] that applied a non-invasive positive pressure ventilation (NPPV) simulation program to general ward nurses in South Korea, as well as another study [28], which conducted a simulation of chronic obstructive pulmonary disease and congestive heart failure. The present study included the process of encountering various problem situations that occurred in mechanical ventilation nursing through VR simulation and solving them through nursing performance. Therefore, these processes may have given nursing students the expectations and beliefs that they successfully performed a certain task. Given the study results [29, 30] that simulation education improved the objective nursing performance of learners and learning self-efficacy by forming an active and enthusiastic attitude, VR simulation education for nursing students was considered to be an effective method for enhancing their self-efficacy for mechanical ventilation nursing.

Third, the hypothesis, “there would be differences in the clinical reasoning competency for mechanical ventilation nursing between the experimental group, to whom the mechanical ventilation nursing VR simulation program was applied, and the control group,” was supported. This result was consistent with that of a previous study on mechanical ventilation-related HFS for nursing students in the UK, wherein clinical decision-making was found to be significantly improved in students, along with an improvement in their critical thinking, noticing, interpreting, reflecting, and responding capabilities [31]. This study supported the results of a study by Salameh et al. [31], which reported that given the increased need for mechanical ventilation due to the current COVID-19 pandemic, providing nursing students with situations requiring mechanical ventilation for patients allowed them to gain real-world clinical experiences that they had not faced before, which suggested that these experiences could be effective in improving nursing students’ clinical reasoning competency.

Fourth, the hypothesis, “there would be differences in the learning immersion between the experimental group, to whom the mechanical ventilation nursing VR simulation program was applied, and the control group,” was supported. This was consistent with the results of a previous study [32] that reported that the use of VR increased motivation for learning in the learners. For education to be delivered more effectively, we recommend employing learning materials and methods that can increase learning immersion, in parallel with simulation education for critical care nursing.

Fifth, the hypothesis, “there would be differences in the learning satisfaction between the experimental group, to whom the mechanical ventilation nursing VR simulation program was applied, and the control group,” was supported. This was consistent with a study conducted on 27 RN-BSN nursing students in the United States, where the satisfaction of the experimental group was higher than that of the control group [33]. In addition, it was also consistent with another study [11] that applied a VR chemoport-embedded nursing education program based on a mobile head mounted display and reported a significant difference in practice satisfaction between the intervention and control groups. This may have been since the educational program developed in this study considered the learning needs of the participants and tried to maximize the educational elements by realistically implementing scenarios and clinical situations with clinical and educational validity. High satisfaction with simulation education was found to improve motivation and, consequently, clinical performance [34], which confirmed that the program developed in this study enhanced students’ competencies.

The COVID-19 pandemic increased the social demand for mechanical ventilation nursing competency [31]. However, it was difficult to reproduce mechanical ventilation nursing for patients who were critically ill with the existing educational methods. In particular, it is thought that the VR simulation program will enable learning without being affected by the time and place. This study had a limitation. It was not possible to randomly assign the participants into the intervention and control groups due to the occurrence of close contact with or confirmed cases of COVID-19. In contrast, the control group received only video lectures. Therefore, it is necessary to compare the simulation education program and the VR simulation education program in the future. In future research, we suggest identifying the long-term intervention effect of a longitudinal study design to predict the lasting effects of the VR program on mechanical ventilation nursing.

## Conclusion

The mechanical ventilation nursing education program using VR simulation is expected to train competent nurses with better clinical judgment in nursing situations requiring mechanical ventilation for patients who are critically ill with COVID-19. This will ultimately contribute to ensuring the safety of such patients and promoting the best possible health in infectious disease situations, thereby contributing to building trust and respect between nurses and patients.

## Data Availability

The datasets generated and/or analysed during the current study are not publicly available because of the potential for compromising student privacy but are available from the corresponding author on reasonable request.
